# Bilateral blunt carotid artery trauma associated with a double lower thoracic spine fracture: a case report and review of the literature

**DOI:** 10.1186/1757-1626-1-420

**Published:** 2008-12-25

**Authors:** Dimitrios S Evangelopoulos, Michalis Athanasakopoulos, Konstantinos Kokkinis, Dimitrios Korres, Spyros G Pneumaticos

**Affiliations:** 1 C' Orthopaedic Department, University of Athens, "KAT" Accident's Hospital, Athens, Greece; 2 Department of Radiology, "KAT" Accident's Hospital, Athens, Greece; 3 Dimitrios Stergios Evangelopoulos, Friebourgstrasse 63, 3008, Berne, Inselspital, Berne, Switzerland

## Abstract

**Background:**

A 26 year old female suffering from a double fracture of the lower thoracic spine, as a result of a car accident, was referred to the Spine Unit of our Department. No neurological deficit was detected during clinical examination. Due to the fracture's instability, posterior stabilization was performed.

**Case presentation:**

Postoperatively, the patient presented a decrease in Glasgow Coma Scale (GCS) combined with motor and sensory deficit from the right upper and lower extremities. Cerebral ischemia was diagnosed, secondary to distal emboli from the right and left internal carotid arteries bilaterally.

**Conclusion:**

Patients' neurological deficits eventually resolved after conservative treatment and anticoagulation therapy.

## Background

Although general trauma is not a common cause of stroke, head and neck trauma associated with cerebrovascular injury is listed as the most frequent cause of stroke in adolescents and young adults [1,2]

Injury of the extracranial carotid or vertebral artery, with associated cervical spine fractures, is a rare but a well documented entity. The first report of an internal carotid artery thrombosis by a non-penetrating neck trauma occurred in 1872 [3]. There have been several reports of patients having pathology in one or more arteries as a result of axial fractures after motor vehicle accidents. More specifically, blunt carotid artery trauma represents only 3% of all carotid artery injuries, but 42% of reported cases have been associated with severe neurologic deficits [1].

We present the case of a patient with blunt carotid trauma bilaterally, combined with double fracture of the lower thoracic spine. To our knowledge, such a case has not yet been described in the literature.

## Case presentation

We present a case of a 26 year old female with a fracture of the 8^th ^and 12^th ^thoracic vertebrae, subsequent to a motor vehicle accident. Upon admission, physical examination revealed a hematoma on the face, and the upper extremities. Glasgow Coma Scale (GCS) was 15/15 and the pupils were equal and reactive to light. The neck was not swollen and no hematomas were observed. Carotid arteries were palpable. Airway was unobstructed and breathing was satisfactory. The patient was hemodynamically stable. The abdomen was soft and non tender. During rectal examination the sphincter tone was normal and no blood was detected. Neurological examination did not reveal any focal deficit.

Imaging studies revealed fractures of the 8^th ^and 12^th ^thoracic vertebrae. Furthermore, she suffered of a fracture of the distal end of the right radius and a type III acromioclavicular separation of the left side (1).

**Figure 1:**
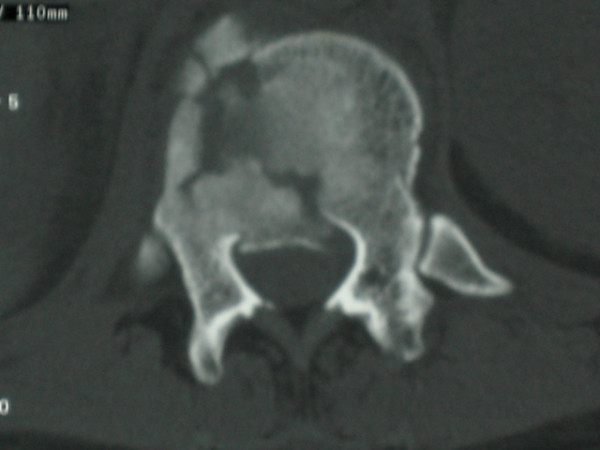
**CT of spinal fracture**.

**Figure 2:**
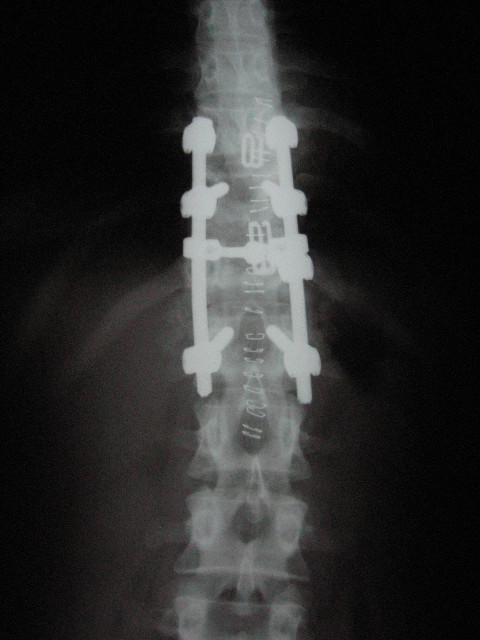
**AP x-rays post-stabilization**.

**Figure 3:**
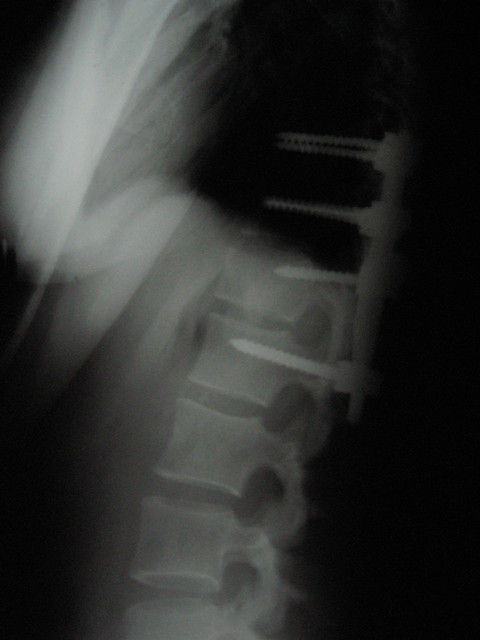
**Lateral x-rays post-stabilization**.

Following appropriate preoperative work up, posterior segmental instrumentation was performed for stabilization of the T12 fracture with the use of pedicle screws. Intraoperative neurophysiologic monitoring was utilized, without any change in the baseline signals (Fig. 2, 3).

Postoperatively, she was lethargic and confused. The patient also developed a right sided hemiparesis. Since it was estimated that the change in the patient's neurological status was of central origin, a new CT-scan of the head was performed as well as a triplex of the carotid arteries. Neither study demonstrated any pathology.

Subsequently, an MRI of the head was performed. The later revealed the presence of a cortical infract at the area of the paracentral lobe as well as a high signal intensity at T2W image and low signal intensity at T1W image in the left caudate and left lentiform nuclei, indicating ischemia in the above mentioned anatomical areas. Pathological signal intensity, indicative of ischemia, was also detected at the anterior portion of the right lentiform nucleus. No other lesions were detected in the areas of brain stem, cerebellum or ventricular system.

**Figure 4:**
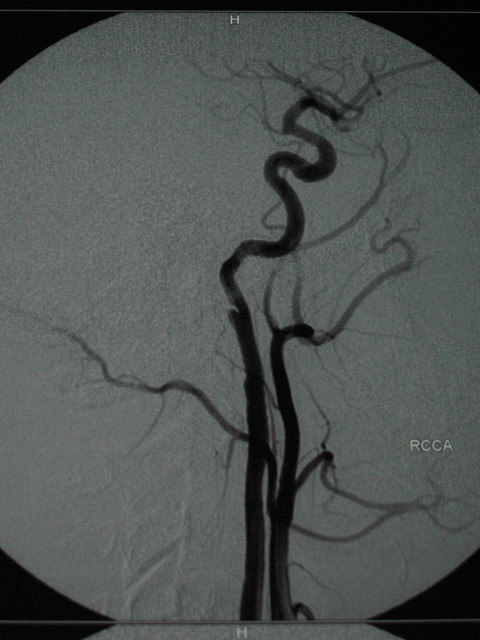
**MR angiography**.

**Figure 5:**
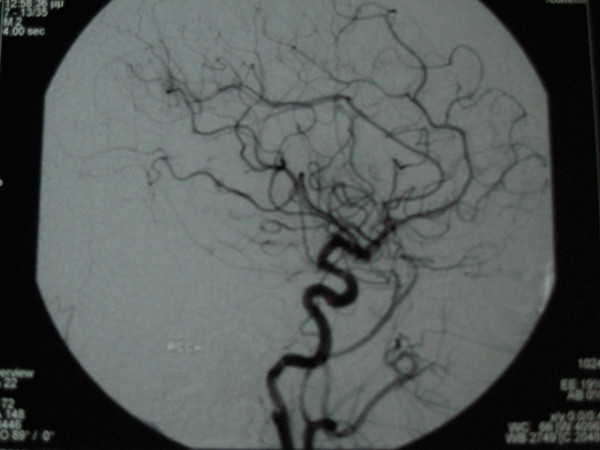
**MR angiography**.

**Figure 6:**
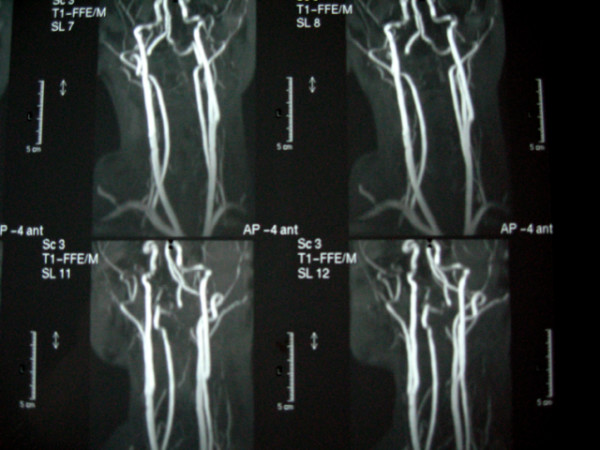
**DS angiography (RCCA, LCCA)**.

**Figure 7:**
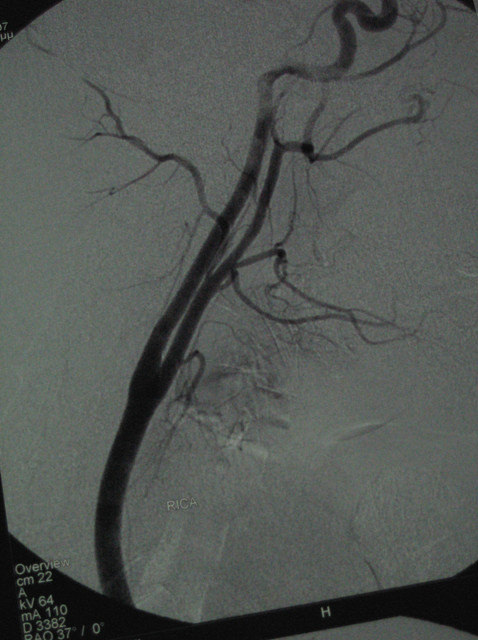
**DS Angiography 6 weeks post trauma (RCCA, RICA)**.

Further evaluation included Digital Subtraction Angiography (DSA) and MR imaging which demonstrated a dissecting aneurysm at the subpetrous portion of the internal carotid arteries bilaterally. No lesions were detected in the endocranial vessels and in the vertebral arteries (Fig 4, 5, 6, 7).

The patient was placed in anticoagulation therapy with intravenous heparin, according to BCI (Blunt Carotid Artery Injury) protocol [4], aiming for an APTT of 40–50. Intravenous anticoagulants were gradually substituted by per os administered Coumadin for at least 3 months [1,5,6]. During the hospital stay, the patient's neurological function improved. At her latest follow up, six months post op, the patient did not demonstrate any motor or sensory deficit in her upper and lower extremities with persistence of brisk deep tendon reflexes in the right side. She is fully ambulatory and is able to perform her activities without difficulty.

## Discussion

According to Crissey et al [7], four types of craniocervical injuries may produce traumatic thrombosis of the internal carotid artery: a) direct blows to the anterior neck in the elderly leading to fracture of an atheromatous plaque at the carotid bifurcation, b) blows at the side of the head in youngsters leading to hyperextension, rotation or lateral flexion, stretching the internal carotid artery across the C3 vertebra. These are usually a result of a major impact, such as violent assaults or motor vehicle accidents, c) blunt intraoral trauma, mainly concerning children and d) basilar skull fracture leading to thrombosis of the intrapetrous portion of the internal carotid artery. However, neither the mechanisms nor the associated injuries can accurately predict the type of lesions as stated by Zelenock et al. [8] since no consistent pattern of associated injuries could be established. According to Yamada et al. [9], the delayed neurological deficits are only safe facts leading to diagnosis, since only 10% of patients present symptoms of transient ischemic attacks or stroke on admission.

Since the risk of a delayed stroke is very high, great importance is given by many authors in finding ways of early diagnosis that would allow the early beginning of anticoagulation or interventional therapy [10-12]. Mears et al. [13], in 1988, reported a case of a blunt carotid artery trauma with neurological deficit and a negative CT-scan of the head, as a result of a blunt cervical trauma. This case resembles to our case in the fact that CT-scan of the head was negative. At the same time, Mooney et al. [14], presented the case of a delayed hemiparesis following a non penetrating carotid artery trauma.

Martin RF et al. [15], in 1991, reported a series of eight patients from a ten-year interval who sustained blunt injuries to the carotid arteries. Six of them (75%) suffered a hyperextension injury or had a cervical spine fracture or both. From those patients, five were treated conservatively. No patient died and seven of eight made a complete neurological recovery or remained asymptomatic. The author concludes that one should suspect such a lesion when dealing with hyperextension injuries, with cervical spine fractures or with patients whose neurological deficits can not be explained by intracranial trauma. Moreover he proposes the conservative treatment for small intimal flaps or dissections in asymptomatic patients.

Cornacchia et al. [16] agree that this injury is associated with cervical spine fractures and conclude, that as many as 40% of the reported cases have permanent neurological deficit. According to the authors, cerebral angiography remains the diagnostic gold standard but other modalities, such as transcranial Doppler and MRI angiography may also be of great importance.

Nevertheless, the aggressive angiographic screening in order to prevent the stroke has not been sufficiently tested. Risk factors for BCI (blunt carotid artery injury) remain numerous, non-specific and not always identifiable prior to the neurologic event [17,18]. Bfill et al. [19,20] in their series, demonstrated that 20% of patients with BCI had no associated injuries and were discovered not because they underwent early angiographic screening but because they developed early neurologic symptoms. The authors, in their effort to group most of the risk factors, concluded that risk factors for BCI were younger age, GCS ≤6, diffuse axonal brain injury, head injury, petrous bone fracture, Lefortt II or III fracture, chest injury and abdominal injury. The authors believe that the most common mechanism of injury is that of hyperextension and contralateral rotation since in this way the upper portion of internal carotid artery is stretched along the anteriorly placed lateral articular processes and pedicles of the upper cervical spine.

Velmachos et al. [21], also announced several possible risk factors for BCI, such as basilar skull and midface fractures, cervical fractures, severe blow to head and neck, broken helmet, neck seat-belt contusion etc. Nevertheless, the author believes that a widely applied, resource-consuming screening program may increase the rate of early diagnosis of BCI, but the improvement in outcome will remain uncertain. Therefore, according to the author, a cost-effectiveness analysis is necessary for trauma surgeons to accept such an aggressive protocol as the standard of care.

On the other hand, Friedman et al. [22], in his study for vertebral artery injury after cervical spine trauma, states that such lesions as determined by MR angiography are common but they remain clinically occult and only a small percentage of patients may suffer devastating neurological complications. According to the author, noninvasive assessment of the vessels by means of MR imaging should be an integral part of the evaluation of the acutely injured cervical spine.

## Conclusion

In our case, no cervical trauma was present. Apart from the thoracic spine fractures T8 & T12, no other spinal fracture were detected. The patient had a GCS of 15/15 upon presentation and no seat-belt sign or other risk factors were present. The only associated injury involved a grade III acromioclavicular separation with mild hematoma involving the right shoulder.

To our knowledge, a case of bilateral internal carotid artery injury associated with fractures of the upper thoracic spines' spinal processes and T8 and T12 vertebrae is not described in the literature. It is important however to keep in mind that polytrauma patients may have suffered occult injuries which the treating physician is not always easy to recognize. One has to investigate the cost effectiveness of extensively evaluating such patients with numerous examinations in the absence of clinical findings, prior to developing a protocol as the standard of care for such patients.

## Abbreviations

CT: Computer Tomography; MR: Magnetic Resonance; BCI: Blunt Carotid Injury; GCS: Glasgow Coma Scale; DSA: Digital Subtraction Angiography

## Consent

Written informed consent was obtained from the patient for publication of this case report and any accompanying images. A copy of the written consent is available for review by the Editor-in-Chief of this journal.

## Competing interests

The authors declare that they have no competing interests.

## Authors' contributions

EDS treated the patient and wrote the manuscript, AM treated the patient and collected the data, KK performed CT-scans, MRI and DS Angiography, KD revised the manuscript, PSG treated-operated the patient and reviewed the manuscript.
